# China: concurring regulation of cross-border genomic data sharing for statist control and individual protection

**DOI:** 10.1007/s00439-018-1903-2

**Published:** 2018-07-16

**Authors:** Yongxi Chen, Lingqiao Song

**Affiliations:** 1 Faculty of Law, The University of Hong Kong, Hong Kong, Hong Kong; 2 Center of Genomics and Policy, McGill University, Montreal, Canada

## Abstract

This paper reviews the major legal instruments and self-regulations that bear heavily on the cross-border sharing of genomic data in China. It first maps out three overlapping frameworks on genomic data and analyzes their underpinning policy goals. Subsequent sections examine the regulatory approaches with respect to five aspects of responsible use and sharing of genomic data, namely, consent, privacy, security, compatible processing, and oversight. It argues that substantial centralised control exerted by the state is, and would probably remain, the dominant feature of genomic data governance in China, though concerns of individual protection are gaining momentum. Rather than revolving around a simplistic antinomy between privacy preservation and open science, the regulatory landscape is mainly shaped by the tension between government desires for national security, state competitiveness, and public health benefits.

## Introduction

Since its initial participation in the Human Genome Project in the 1990s, China has actively promoted genomic research for the purposes of medical care improvement and scientific advancement (Zhan and Qian 2016). The regulation of the use and sharing of genomic data, however, is complex as it derives from diverse policy concerns. The Chinese government has strengthened the protection of individuals’ interests pertaining to this data, such as privacy and personal dignity, but maintains the policy thrust on promoting national interests associated with genetic resources.

This paper reviews the major legal instruments and self-regulation rules that bear heavily on the cross-border sharing of genomic data in the People’s Republic of China (PRC), and maps out their underpinning rationales. It first delineates three overlapping policy frameworks based on different perceptions of genomic data. Subsequent sections examine the regulatory approaches with respect to five aspects that are normally addressed under international policy frameworks for responsible use and sharing of genomic data (Knoppers 2014; Sugano 2014).

## Compartmentalized regulatory frameworks on genomic data

**Fig. 1:**
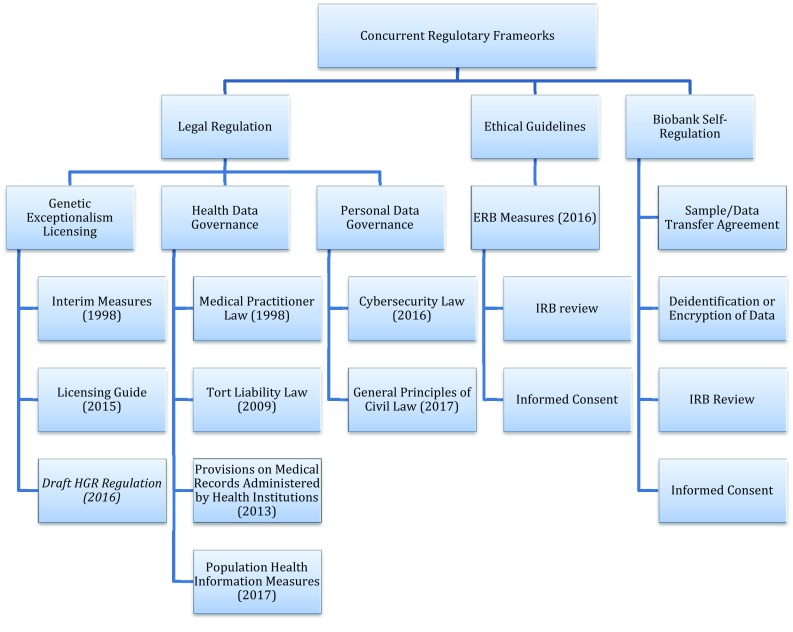
Concurrent regulatory frameworks on genomic data sharing

It is not easy to navigate through the labyrinth of China’s legal instruments of variable legal status that bear on the sharing of genomic data. Identifying major regulatory frameworks that address divergent policy concerns can serve as a compass to relevant instruments, and help to understand the fast-changing governance approaches to genomic and health data in China. The frameworks can be analytically divided into three, each stemming from a different perception of the nature of genomic data and its political and/or social implications (see Fig. 1).

### Administrative licensing concerning human genetic resources

**Fig. 2:**
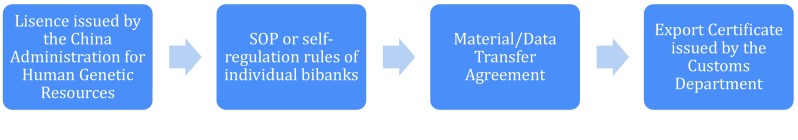
Outbound process for cross-border genetic sample or data sharing

Administrative licensing is the first and foremost phase for genomic data to be legally transferred overseas. The licensing framework treats genetic materials as unique resources for the nation’s collective good and places them under stringent state control, which can be regarded as a “genetic exceptionalism” model (Joly et al. 2017).^1^ According to the foundational instrument, *Interim Measures of Human Genetic Resources* (PRC 1998a) (*Interim Measures* hereafter), human genetic resources refer to *genetic materials*, such as human tissues, cells, blood specimens, preparations, or recombinant DNA constructs that contain the human genome, genes or gene products, and *information concerning such materials* (emphasis added by the authors),^2^ thus encompassing genomic data [Art. 2]. The collection, storage, and export of human genetic resources are all subject to the government’s prior approval and the standing oversight by the China Administration of Human Genetic Resources (CAHGR) [Arts. 4 & 7], which is under the joint supervision of the Ministry of Science & Technology (MOST) and the Ministry of Health (MOH). With the *Administrative Licensing Law* coming into force in 2004, examination and approval have been undertaken through the administrative licensing process (PRC 2003). Figure 2 illustrates the process for cross-border sharing of genetic samples and data.

The *Interim Measures* stipulates that domestic R&D institutions enjoy the exclusive possession right over information about human genetic resources, in particular data concerning important pedigrees and genetic resources in specified regions [Art. 17]. It forbids the extraterritorial provision of such information and resources without authorization. The only lawful means for an overseas entity to access genomic data is through an international collaboration project with a Chinese institution, which should further apply for approvals from both the authority that governs the institution and the CAHGR [Art.11]. Apart from the consent requirements (to be discussed later), the conditions for approval stress that the ownership of intellectual property should be clear and the sharing of benefits reasonable and that the overseas collaborator should have relatively strong research and development capacity and advantages [Arts. 12 & 13].

In view of the brevity of the *Interim Measures*, the MOST drafted a more comprehensive *Regulation on Human Genetic Resources* (*draft HGR Regulation* hereafter) in 2012 (Legislative Affairs Office of the State Council 2012). 4 years later, the State Council released a revised draft for public comment (Legislative Affairs Office of the State Council 2016). Licensing under the *2016 draft HGR Regulation* does not apply to the handling of genetic resources for clinical diagnosis, and focuses on R&D activities of overseas entities or domestic institutions with overseas investments. In addition to conditions provided under the Interim Measures, applicants are required to justify the reasonableness in engaging in international collaboration and export of genetic resources [Art. 17(3)]. Most importantly, the collaboration and/or export will be rejected when they “may jeopardize national security, national interests or public security” [Art. 19 (7)]. As the regulation is still pending, the MOST has incorporated most requirements of the draft into a new “*Licensing Guide*” that refines the licensing conditions, which took effect on October 1, 2015 (MOST 2015).

This robust state control is mainly grounded on biosecurity considerations and the desire for national competitiveness. Anxiety over bio-piracy was triggered by media coverage of the Anhui incident in 1997. Two occupational epidemiologists affiliated with Harvard University collected blood samples for a genetic project from over 16,000 Chinese peasants in Anhui Province without appropriate informed consent, and were subsequently disciplined by the university (Lawler 2002; Chen et al. 2015). Prominent Chinese scientists, in particular Chinese geneticists, called for the government to undertake actions to protect the nation’s genetic resources against foreign exploitation (Fu 2018; Zhang and Zheng 2017). The enactment of the Interim Measures was a prompt response.

The *2016 draft HGR Regulation* further declares “safeguarding national security” as one of its legislative purposes, with biosecurity as a core element of national security. It also emphasizes equality and mutual benefits in international collaboration. According to the MOST, domestic genetic materials concerning ethnic groups, pedigrees, and typical diseases are strategic resources for life science as well as biomedical technologies and industries, and having control over these materials will significantly contribute to a country’s position among the stiff international competition in those areas (MOST 2016). The *draft Regulation* further specifies that overseas collaborators shall ensure that Chinese collaborators have substantially participated in the R&D activities [Art. 16]. This move may relate to the authority’s concern over the practices in recent years that domestic researchers play a marginal role in publishing findings that are based on Chinese genomic data shared with overseas collaborators (Yuan 2017). Echoing the repeated warning against illegal seizures of genetic resources by foreign entities, the drafters identify in particular cross-border data transfer as a new and covert means of seizure. This position is in a distinctive contrast with international consensus on the imperative of genomic data sharing, as recognized under the *Bermuda Principles* (HUGO 1996), the *Fort Lauderdale Agreement* (2003), and initiatives of building interoperable rules of sharing, such as the *Framework for Responsible Sharing of Genomic and Health-Related Data* of the Global Alliance for Genomics & Health (GA4GH 2014).

Based on the same rationale, the MOST launched nationwide audit campaigns in 2011 and 2013 to identify sino-overseas projects that are unauthorized or uncompliant with state policies (Feng and Huang 2018). According to the number released by the CAHGR, more than 100 transnational projects involving human genetic resources have been approved in 2017 alone. The frequency of the license approval has shifted from a quarterly to semimonthly basis resulting from an attempt to meet the voluminous projects awaiting administrative approval (MOST 2018). While supporting international collaboration, the CAHGR has statutory power to revoke approved licenses. It is noteworthy that in February 2018, the CAHGR revoked the licenses granted to two high-profile collaborative projects, which concern the Comparative Genetic Study of Psychosis in Han Chinese (between UCLA and Shanghai Jiaotong University) and the Genetic Foundation of Depression in Chinese Women (between Oxford University and Peking University), respectively, and confiscated the exported genomic data (CAHGR 2018). The revocation was made pursuant to the *Administrative License Law*, but no specific reasons were disclosed in the formal decision.

### Health data governance

While genomic data are part of human genetic resources amenable to research, it can also be generated during the provision of health care and hence become health data, falling under the jurisdiction of health authorities. Legal instruments in this jurisdiction are mainly oriented towards protecting interests of the individuals, such as ensuring good clinical care, respecting participants’ autonomy and preserving privacy. The individual-centric values are typically embodied in the *Measures on the Ethical Review of Biomedical Research Involving Human Subjects* (*ERB Measures* hereafter) (MOH 2016).^3^ However, there are also instruments inspired by the need to build up state competitiveness, such as strengthening scientific research and facilitating the medical industry’s growth. The values of collective good are emphasized by the *Measures on Population Health Information* (*PHI Measures* hereafter) (MOH 2014)^4^ and the *Guiding Opinions on Promoting and Regulating the Application of Big Medical and Health Data* (General Office of State Council 2016).

The *PHI Measures* is currently the general instrument that regulates the collection, management and use of “population health information”, which refers to information generated during healthcare services by health institutions, covering both population-level summary data and individual-level identifiable data [Arts. 3 & 15]. While facilitating the nationwide connectivity and interagency/inter-institutional sharing of population health information, it categorically prohibits the storing of such information in overseas servers [Art. 10]. This provision affects the means of genomic data sharing according to a compartmentalization of the data generator. Genomic data generated by health institutions (e.g., hospitals, clinical test labs, and disease control centers) are not allowed to be stored in overseas institutions or transferred to a cloud-computing environment that is not based in China and that serves many international biobanks or research consortia, but can only be accessed from Chinese servers subject to the technical and policy control of individual health institutions. For genomic data generated by entities other than health institutions (e.g., research institutions, biobanks not affiliated with hospitals, and providers of direct-to-customer services), it may be transferred across the border if the CAHGR so approves.

### Personal data governance concerning privacy and cybersecurity

Insofar as genomic data contains personally identifiable data (including reversibly de-identified data), it is subject to a variety of legal instruments over the processing of personal data, whose policy goals include, most importantly, safeguarding privacy and cybersecurity. Due to the lack of a general law for personal data protection as well as a common statutory definition of privacy, sectoral instruments offer remedies to data privacy that depend on and differ between contexts (e.g., consumer data and telecommunication data). The obscure border of data privacy has hindered the regime of freedom of information which could otherwise enable re-use of government information for social progress (Chen 2015). The incoherence in privacy often leaves data users uncertain about the scope and manner of lawful data sharing. Major instruments that affect genomic data processing will be further analyzed in “Privacy”.

In addition, lawmakers have recently tended to associate data protection with “cybersecurity” which emerges as an overriding state concern. Enacted in 2016, the *Cybersecurity Law* is now the highest level instrument that governs data transmitted and processed online. Apart from incorporating important guarantees of data privacy, the law emphasizes “cyberspace sovereignty” and national security, imposing on data users comprehensive obligations with respect to network operation and control of data content (PRC 2016). Article 37 has a particularly significant impact on the transfer of genomic data. It prohibits “personal information or important data collected and generated within China by *critical information infrastructure operators*” (emphasis added by the authors) from being stored overseas. Where necessity exists in providing such data to overseas parties, domestic operators shall undergo security assessment in accordance with the rules formulated by the Cyberspace Administration of China (CAC) and relevant departments of the State Council. As the rules are currently pending before the State Council, it remains unclear as to whether genomic data stewards inside China fall within the scope of “critical infrastructure operator” and what security assessment measures they should comply with (Livingston and Greenleaf 2017). Overall, the *Cybersecurity Law* strengthens the restriction over cross-border genomic data sharing, and its state-centric policy goals echo those underpinning the “genetic exceptionalism” licensing framework.

With the major types of regulation unravelled, the following sections review the regulatory approaches pertaining to the specific dimensions of genomic data sharing.

## Informed consent

As one of the most fundamental ethical principles, informed consent plays an essential role in protecting the individual’s autonomy and privacy in China. Consent is recognized as a legal requirement for medical treatment and for the disclosure of medical information according to the *Tort Liability Law* [Art. 62]. Informed consent is also widely recognized as an ethical principle in the fields of biomedical research and clinical trials (Yu and Li 2014). According to the *Interim Measures*, informed consent is a precondition for any project that intends to make use of Chinese human genetic resources. Marked by a typical collectivist philosophy, informed consent is not only required from the participants, but also from their family members [Art. 12]. However, there is no further provision specifying the scope of the family members, which renders uncertainty in implementing the family consent in practice.

Compared to the *Interim Measures*, the *2012 draft HGR Regulation* incorporates more detailed provisions regarding the consent form. It stipulates that consent should be given to the purpose of the research, potential risks and benefits, privacy protection, the right to withdrawal, and voluntary participation. To ensure that participants reach a full understanding of the consent, the *draft Regulation* requires that the language of the content corresponds with the literacy level of the participant [Art. 14]. The *2016 draft Regulation* replaces these detailed provisions with a requirement that the standard form and text of informed consent should be approved by the institutional review boards (IRB) of both domestic and overseas collaborators [Art. 18], thus leaving latitude for IRBs to regulate the form and content of informed consent.

As a regulation largely in compliance with the Declaration of Helsinki, the *ERB Measures* establishes the principle of informed consent as one of the basic ethical principles [Art. 18(1)]. It broadly applies to medical institutions of all tiers that carry out the biomedical research involving human subjects [Art. 2]. It requires voluntary informed consent before the research begins, leaving room for oral consent with the condition that supporting records are available [Art. 33]. Like the *2012 draft HGR Regulation*, it requires that appropriate language be used for the informed consent [Art. 35]. More importantly, Article 38 stipulates situations in which the participants shall be re-contacted including: changes made to the proposal, scope or content of the research, secondary use of the identifiable diagnostic and clinical samples, and secondary use of identifiable human biological samples and associated clinical record in a biobank. However, inconsistent with the family consent mentioned in the *Interim Measures*, the *ERB Measures* are silent about whether it is only participant or family members should also be re-contacted. Article 39 endorses the exemption from informed consent by specifying the situation, where the participant has provided the informed consent and agreed to the usage of samples and their derived information for all biomedical research. IRB approval is required in this scenario. These provisions indicate the Chinese government’s attempt to incorporate different types of consent form to meet the new demands raised by biobanks and genomic researchers. However, the instrument lacks specific provisions on the consent for transnational genomic data sharing, leaving a big gap to bridge (Wang et al. 2014).

More specific rules concerning informed consent in cross-border genomic data sharing can be found in the policies of large-scale Chinese biobanks such as the China National Genebank (CNGB). There are roughly 49 biobanks throughout China until 2015, among which the large-scale ones, such as the CNGB and China Kadoorie Biobank (CKB), have relatively comprehensive data sharing policies (Zhang et al. 2015). Each biobank is entitled to draft its own standard operating procedures (SOP) or data sharing policy in accordance with the *Interim Measures* and other legal instruments. In 2017, the CNGB together with the Huazhong University of Science and Technology drafted the *Ethical Guidelines and Regulatory Norms for the Sharing of Biobank Samples and Data* (*draft Sharing Guidelines* hereafter) for adoption by biobanks nationwide. This document refers widely to various international rules such as ISBER Best Practices (2012) and UK Biobank Ethics and Governance Framework (2007). (CNGB 2018). Its main purpose is “to promote sample and data sharing, to accelerate scientific development, and to protect the rights and benefits of the donors” [Sec. 1].

Under this self-regulation document, informed consent is a precondition for the collection of samples and derived personal information [Sec. 6.3]. In terms of the export of personal data, individuals should be informed of and further agree to the purpose, scope, content, recipients, and the receiving country or regions of the information. Where the personal information of minors is involved, consent from his/her legal guardian is also required [Sec. 5.3]. In addition, it specifies the possible situations for acquiring a group consent form, where the sample or data collection focuses on a specific group [Sec. 4.1]. The *Sharing Guidelines* indicates that considerable weight has already been put on informed consent by Chinese biobanks in facilitating global genomic data sharing (CNGB 2017). They have attempted to incorporate the globally recognized standard to meet new trends of international genomic data sharing, which is reflected in their own industry guidance (CNGB 2017). However, these integration initiatives are still in their infancy and need to be further adapted to the Chinese reality.

## Privacy

Privacy is a principal concern underpinning convergent international codes of conduct for genomic data sharing (Knoppers et al. 2014). Yet, no specific Chinese legal instrument has been enacted for preserving privacy in the context of cross-border genomic data sharing. Privacy protection is provided sporadically under sectoral legislation. Besides, biobanks such as the CNGB are embarking on stipulating more specific privacy or confidentiality obligations in their institutional rules. This section reviews major legislation, whose provisions bear on genomic data privacy as well as privacy rules prepared by the CNGB.

As discussed in “Compartmentalized regulatory frameworks on genomic data”, protection of genomic data under the *Interim Measures* is mainly based on the perception of Chinese human genetic materials as the state’s collective resource. Therefore, it is not surprising that its text barely mentions privacy. The subsequent *Licensing Guide* does not explicitly refer to privacy protection either (Chen et al. 2015). The *2016 draft HGR Regulation* makes progress in forbidding disclosure of privacy of genetic resource donors, but sanction is imposed only when the divulgence causes “severe adverse social impacts” [Art. 32]. This implies that genomic privacy is still incidental to the collective interest-centric regulation [Art. 38].

The protection of genomic privacy has to be sought from a non-genetic exceptionalism perspective. A general right to privacy is stipulated in the *Tort Liability Law* [Art. 2] (PRC 2009) and the recent *General Principles of Civil Law* [Art. 110] (PRC 2017), but no statutory definition of privacy has been provided. Under the Chinese civil law doctrine, information/data privacy constitutes a part of privacy (Wang 2011). Data privacy has become concurrently protected under legal instruments that regulate the processing of personal information. Notably, the *General Principles of Civil Law* explicitly prescribe the protection of personal information in addition to the right to privacy. This new law prohibits “any illegal collection, exploitation, processing, transmission, trading, provision or disclosure of an individual’s personal information” [Art. 111]. More comprehensive legislative protection of personal information is offered by the *Cybersecurity* *Law* (2016), though the latter is widely regarded as being national security-based instead of individuals’ rights-oriented (Parasol 2017; Loper 2018). It stipulates that “[Information] network operators collecting and using personal information shall abide by the principles of legality, propriety and necessity; make public the rules for collection and use, explicitly stating the purposes, means, and scope for collecting or using information, and obtaining the consent of the person whose data is gathered” [Art. 41]. To make the broad provisions of the *Cybersecurity Law* more operable, a series of implementing rules and guidelines have been made, among which the *Personal Information Security Specification* is most relevant to genomic data privacy (NISSTC 2017b). Being a non-binding national standard made by the National Information Security Standardization Technical Committee (NISSTC, which is under the Standardization Administration of China), the *Specification* expresses the best practices recommended by the cybersecurity authority. It delineates the scope of sensitive information within which genetic information and family history fall. This move indicates that policy makers have considered the implications of genetic information in cybersecurity. In addition, the *Specification* has remarkably attempted to align itself with globally recognized privacy protection measures by providing for de-identification [Sec. 6.2], anonymization [Sec. 3.1.3], template of privacy policy [Sec. 5.6], and, more generally, manners of sharing or transferring personal information [Sec. 8]. Genetic information was also mentioned in the Supreme People’s Court judicial interpretation concerning the civil liabilities of information network operators (SPC 2017), which prescribes that the court should find tort liability to be born by “the internet users or internet service providers disclose individuals’ genetic information, medical record, diagnosis result […] and other personal information through the internet with damages to individuals”.

Data privacy of patients is also governed by a set of instruments that mainly regulate health care activities. Adopted in the early 1998, the *Medical Practitioner Law* specifies that medical practitioners have the duty to protect patients’ privacy, and imposes disciplinary sanctions and administrative penalties for severe privacy infringement [Art. 22] (PRC 1998b). In response to the wide application of the electronic health record, the MOH issued the *Basic Norms on Electronic Medical Records* (2010) to establish stratifying levels of access to medical records by the personnel in health institutions [Art. 16]. The MOH also amended the *Provisions on Medical Records Administered by Health Institutions* (2013), providing that no person other than practitioners engaging in medical care shall inspect patients’ records without prior authorization [Art. 6]. The *PHI Measures* further refers to privacy preservation under multiple articles [Arts. 2, 5, 6, 13 and 16]. Civil liabilities are imposed on breaching patients’ privacy or disclosing medical records without consent of the concerned patient, which is stipulated by the *Tort Liability Law* (2009) [Art. 62]. Overall, these instruments protect genomic data as included in medical or other records held by health institutions against disclosure, but none takes into account the trend of global genomic data sharing, hence leaving a gap to be filled by other regulatory frameworks.

In addition to the above-mentioned legal protection of privacy, the *ERB Measures* stresses privacy protection as one of the fundamental ethical principles (MOH 2016). It outlines basic privacy protection for biomedical research including international projects involving human genetic resources. Researchers should “…safeguard the subject’s privacy; fully inform the subject of the storage, usage, and security measures of the personal information; and not disclose the personal information of the subject to third parties without authorization” [Sec. 18.4].

As mentioned in “Informed consent”, CNGB is taking active moves in regulating genomic data sharing. The *draft Sharing Guidelines* stipulates “privacy protection and data security” as one of the fundamental principles [Sec. 2.2]. Encrypted or anonymized methods are required to protect the donors’ privacy [Secs. 4.2 & 5.2.2.2]. Moreover, IRB review is mandatory for all project involving human samples or associated data [Sec. 4.4]. Sharing can be enabled only within the Chinese territory unless otherwise licensed by the CAHGR [Sec. 5.2.3.4]. In view of this, biobanks’ data sharing policies are limited or restricted by the Interim Measures. The draft Guidelines further lays out the concrete rules for cross-border sample and data sharing [Sec. 5.3] and stipulates that data can be shared only for the purpose of research [Sec. 7.1]. In addition, the *CNGB Security Rules*, which applies only to the CNGB itself, contains similar measures that protect the participants’ privacy and stress the importance of IRB review [Chapter 6] (CNGB 2017).

## Security

The *Licensing Guide* prescribes that the applicant (i.e., the domestic collaborator) should have established a dedicated system for managing genetic resources and that the foreign collaborator should have the capacity for conducting the research (which includes, understandably, appropriate security arrangements). The regulation thrust is to prevent international collaboration from endangering national security and/or public security.

The *PHI Measures* imposes more detailed security obligations that can enhance privacy. It requires health institutions that provide genomic data to establish security management systems in accordance with the national regime of graded protection of information security [Art. 16], and maintain a tracing system to ensure real-name identification and access control of users who create, amend, and access such data [Art. 18]. Overseas researchers who are authorized to access genomic data created by Chinese health institutions are thus subject to a stricter security clearance system.

Among the self-regulations of biobanks, the *CNGB Security Rules* exemplifies a fairly comprehensive security arrangement for genomic data sharing, especially in the cross-border context. Its nine chapters stipulate organizational, physical, and technical measures for biosecurity, data security, and confidentiality in the genebank’s operation. Under Chap. 8, which governs the sharing of samples and data for R&D purposes, the sharing should be conducted on a dedicated platform in compliance with various security measures [Arts. 31 & 32], including, in particular, identity verification, tiered access control and the logging of data use for audit [Art. 12]. Data life cycle management strategies and mechanisms for data leakage report and response should also be in place [Arts. 15 & 18]. Overseas users of genomic data should sign a confidentiality agreement with the genebank [Art. 38].

The China Kadoorie Biobank, jointly operated by the Chinese Academy of Medical Sciences and Oxford University, also includes security requirements in its terms of data access for international users. Applicants shall have formal policies and procedures to ensure that the data set is stored securely and used responsibly, with the appropriateness of such safeguards approved by the independent Access Committee (CKB 2014).

## Compatible processing and adequacy

Stemming from the privacy and security requirements, compatible processing and adequacy in data protection in the countries receiving data are two important considerations in cross-border genomic data transfers. Neither the *Interim Measures* nor the *Licensing Guide* provides for compatible processing of genomic data. Given the strict prior approval system, any proposed change in the authorized use of data is to be reviewed by the CAHGR on an ad hoc basis. Thus, for genomic data transferred overseas, any further processing that differs from the purpose for which the data originally shared will not be automatically approved. Likewise, under the *PHI Measures*, genomic data shared by health institutions should not be used or disclosed in a manner that goes beyond the scope of authorization [Art. 14]. Chinese biobanks adopt a similar stance. According to the CKB, except if required by a court order, access will be permitted only for purposes consistent with the aims and ethics of the original study (including the original signed consent). If there is substantial deviation or change in the planned use of the data, further approval will be needed (CKB 2014).

With regard to the issue of adequacy, it is not addressed in the *Cybersecurity Law*, but mentioned in the rules that are being drafted to implement the law. The CAC, the national supervisor of cybersecurity, drafted the *Security Assessment Measures for the Export of Personal Information and Important Data* in 2017 (CAC 2017a), under which all network operators should assess the “cybersecurity environment” in the countries that receive personal information and important data [Art. 8] before exporting such data. A month later, the NISSTC, the entity that sets national standards for information security, drafted the *Guidelines for Security Assessment of Cross-Border Data Transfer* for public comment (NISSTC 2017a). The draft *Guidelines* further specifies that network operators engaging in cross-border transfer of personal information should evaluate the “security risks” in the receiving country’s political and legal environment, which include in particular the gap between the protective level offered by the personal information laws and standards in the country and those in China [Sec. 5.2.6.1; B.3.3.1]. A greater gap may add to the overall security risk and entails obligations for the operator to take measures to mitigate the risk or adjust the data export plan [Sec. 4.6]. Though being a non-binding standard, the *Guidelines*, once issued, can be referred to by the cybersecurity authority when it inspects whether a genomic data steward has exercised due diligence before exporting data. Both the mandatory legal rules and best practices standards on this issue are likely to be settled in the near future.

## Oversight

Oversight mechanisms under relevant laws focus mainly on the monitoring of data use and sanctions for misconduct. According to the *Administrative Licensing Law*, the authority that grants a license has a mandatory obligation to inspect the licensees’ activities under the license and disclose the inspection records to the public [Art. 61]. As regards genomic data sharing, it is the authorities at or above the provincial level in charge of science and technology that conduct the inspection, e.g., examining the data processing site, reviewing relevant materials, and interviewing the concerned personnel [Arts. 28 & 29]. It is noteworthy that a license of international research collaboration can be annulled by the authority ex officio or upon request of the interested parties. A license may be annulled under the following conditions: an applicant who is not eligible or does not meet the statutory conditions has been approved for the license, the licensee obtained the license through cheating or other illegitimate means or “other circumstances under which the license can be annulled in accordance with the law” [Art. 69].

Under the *Interim Measures*, Chinese institutions or individuals that provide human genetic resources to overseas institutions or persons without authorization will be subject to administrative penalties or other legal liabilities, and concerned resources will be confiscated [Art. 21]. The *2016 draft HGR Regulation* further stipulates administrative penalties for overseas institutions which have been licensed to access human genetic resources but commit infractions including, among others: (1) violation of the informed consent principle; (2) breach of the resource donors’ privacy with adverse social impacts; and (3) unauthorized change of the subject, purpose, content, and period of the research project and the scheme of intellectual property distribution. Such institutions will be imposed a fine ranging from 50,000 to 200,000 Chinese yuan (approximately 7800 to 31,200 USD); if the circumstances are serious, they will be forbidden from collecting or accessing human genetic resources for a year and imposed a fine ranging from 200,000 to 500,000 yuan (31,200 to 78,000 USD) [Art. 32].

With regard to genomic data held by health institutions, the *PHI Measures* also provides for regular inspection by health authorities and that the institutions are subject to disciplinary actions which manage health data in violation of this instrument.

The oversight systems established by Chinese biobanks have tended to take an approach common to large international data repositories. The CNGB, for example, designates the IRB to regularly inspect the undertaking of data access projects that it has approved and investigate any adverse events therein. The Committee is authorized to order the suspension or termination of projects that violate the terms of access or engage in unethical behaviours (CNGB 2017). The CKB has established an independent Access Committee to provide oversight and guidance with any access applications that raise a particular issue. Applicants, or collaborators, are required to submit regular progress reports to the CKB Steering Committee. If there is difficulty in completing the planned research, the CKB’s Steering Committee will have the right, after consultation with the Access Committee, to terminate the work if it believes there is little chance that the problem will be rectified. In addition, when data users return the original data sets upon completion of the research, the CKB staff may carry out independent checks and/or validation of the data to ensure the continued data integrity (CKB 2014).

## Future trends

Given the making or amendment of sectoral legal instruments concerning health-related data in the near future, together with the adoption of self-regulations and policies that take international guidelines into account, the regulation of cross-border sharing of genomic data in China will be supported by more precise standards. It is likely to stay compartmentalized and multi-layered, nevertheless.

The progress in sector-specific privacy legislation will help to ease the worry over abuse of data of patients, research participants, and online data in general. This has a cumulative effect on strengthening public trust in broadening the sharing of genomic data for research and clinical trials. That incremental enhancement is, however, weakened by the inconsistent levels of privacy protection under the instruments issued by different authorities governing the essentially same set of data. In addition, consent has gained greater policy weight, but individuals’ control over the process of sharing their sensitive data needs more operable enforcement arrangements.

Policies in favour of scientific data sharing have gained momentum because of the recent adoption of the national strategy in unlocking the potential of big data for technological innovation, industrial growth, and social benefits (State Council 2015). The State Council issued in March 2018 the *Scientific Data Measures* to promote domestic sharing of data generated in publicly funded scientific research (General Office of the State Council 2018). The accompanying explanations refer to foreign biobanks as an inspiring example for maximizing the social benefits of open data (Yuan 2018). For years, Chinese scientists have been advected for government initiatives that overcome hurdles in sharing biomedical data and establish big data infrastructure for efficient research collaboration nationwide. The new *Scientific Data Measures* will probably facilitate further flourishing of biobanks and medical data centers in China. In addition, since December 2017, a streamlined licensing process is in place for genomic data used in multi-sites international clinical trials for obtaining the listing of drugs and medical equipment, which allows the co-investigating institutions to recognize the ethical review results in the principal institution to avoid repeated examination by the CAHGR (MOST 2017).

It is, nevertheless, noteworthy that the substantive restrictions on the cross-border transfer of genomic data for research purposes remain unchanged. A possible tightening is implied by the annulment of two licenses in early 2018 as well as the categorical ban on storing data overseas under the *PHI Measures*. From the central government’s perspective, prioritizing domestic use of genomic data and scrutinizing the distribution of intellectual properties to overseas collaborators are compatible with its objective to gain the leading edge in biomedical research and industries (MOST 2016).

Furthermore, national security continues to be a weighty concern that drives both the licensing framework and the cybersecurity inspection of genomic data. A series of implementing rules of the *Cybersecurity Law* are to be enacted. Under the current draft rules published by the CAC, information systems operated by health institutions and pharmaceutical enterprises belong to “critical information infrastructures” (CAC 2017b), whose operators bear extra obligations of data management, and population health information falls within the scope of “important data”, whose export should undergo careful security assessments (NISSTC 2017a). Both instruments suggest a more stringent inspection of genomic data export by the cybersecurity authority in addition to the examination by the dedicated supervisor of genetic resources.

Overall, statist control is and will probably remain the dominant feature in genomic data governance for years to come. Rather than revolving around a simplistic antinomy between privacy preservation and open science, the regulatory landscape in China is mainly shaped by the tension between the regulators’ desires for national security, state competitiveness, and public health benefits.

## Footnotes

1 The Genetic exceptionalism model treats genetic information different from other types of health or personal information, usually by adopting a special law in regulating genetic information.

2 This enactment was approved by the State Council, and is an administrative regulation whose legal force is inferior only to that of the Constitution and of the laws adopted by the national legislature.

3 This enactment is a ministerial rule whose legal force is inferior to that of laws and administrative regulations.

4 This enactment is a national-level “normative document” whose legal force is inferior to that of laws, administrative regulations and ministerial rules.
