# Correction to: China: concurring regulation of cross-border genomic data sharing for statist control and individual protection

**DOI:** 10.1007/s00439-018-1926-8

**Published:** 2018-08-17

**Authors:** Yongxi Chen, Lingqiao Song

**Affiliations:** 1 Faculty of Law, The University of Hong Kong, Hong Kong, Hong Kong; 2 Center of Genomics and Policy, McGill University, Montreal, Canada

## Correction to: Human Genetics 10.1007/s00439-018-1903-2

The article China: concurring regulation of cross-border genomic data sharing for statist control and individual protection, written by Yongxi Chen and Lingqiao Song, was originally published electronically on the publisher’s internet portal (currently SpringerLink) on 16 July 2018 without open access. With the author(s)’ decision to opt for Open Choice the copyright of the article changed on 16 August 2018 to © The Author(s) 2018 and the article is forthwith distributed under the terms of the Creative Commons Attribution 4.0 International License (http://creativecommons.org/licenses/by/4.0/), which permits use, duplication, adaptation, distribution and reproduction in any medium or format, as long as you give appropriate credit to the original author(s) and the source, provide a link to the Creative Commons license and indicate if changes were made.

The original article was corrected.

